# Designing Health Recommender Systems to Promote Health Equity: A Socioecological Perspective

**DOI:** 10.2196/60138

**Published:** 2025-01-30

**Authors:** Caroline A Figueroa, Helma Torkamaan, Ananya Bhattacharjee, Hanna Hauptmann, Kathleen W Guan, Gayane Sedrakyan

**Affiliations:** 1 Faculty of Technology, Policy and Management Delft University of Technology Delft Netherlands; 2 School of Social Welfare University of California, Berkeley Berkeley, CA United States; 3 Department of Computer Science University of Toronto Toronto, ON Canada; 4 Utrecht University Utrecht Netherlands; 5 Department High-Tech Business and Entrepreneurship Section Industrial Engineering and Business Information Systems University of Twente Enschede, Overijssel Netherlands

**Keywords:** digital health, health promotion, health recommender systems, artificial intelligence, health equity, AI, digital devices, socioecological, health inequities, health behavior, health behaviors, patient centric, digital health intervention

## Abstract

Health recommender systems (HRS) have the capability to improve human-centered care and prevention by personalizing content, such as health interventions or health information. HRS, an emerging and developing field, can play a unique role in the digital health field as they can offer relevant recommendations, not only based on what users themselves prefer and may be receptive to, but also using data about wider spheres of influence over human behavior, including peers, families, communities, and societies. We identify and discuss how HRS could play a unique role in decreasing health inequities. We use the socioecological model, which provides representations of how multiple, nested levels of influence (eg, community, institutional, and policy factors) interact to shape individual health. This perspective helps illustrate how HRS could address not just individual health factors but also the structural barriers—such as access to health care, social support, and access to healthy food—that shape health outcomes at various levels. Based on this analysis, we then discuss the challenges and future research priorities. We find that despite the potential for targeting more complex systemic challenges to obtaining good health, current HRS are still focused on individual health behaviors, often do not integrate the lived experiences of users in the design, and have had limited reach and effectiveness for individuals from low socioeconomic status and racial or ethnic minoritized backgrounds. In this viewpoint, we argue that a new design paradigm is necessary in which HRS focus on incorporating structural barriers to good health in addition to user preferences. HRS should be designed with an emphasis on health systems, which also includes incorporating decolonial perspectives of well-being that challenge prevailing medical models. Furthermore, potential lies in evaluating the health equity effects of HRS and leveraging collected data to influence policy. With changes in practices and with an intentional equity focus, HRS could play a crucial role in health promotion and decreasing health inequities.

## Introduction

### Overview

Health recommender systems (HRS), information retrieval systems that often use artificial intelligence, are an increasingly popular method to provide patient-centric personalized health services [1-3]. HRS predict the relevance of recommendations (eg, for exercise, healthy foods, and mental health exercises) for a given user profile [4]. HRS base recommendations on what the user might like, typically using various sources of data such as personal (eg, age, gender, and socioeconomic status [SES]), health (eg, medical history and health surveys), contextual (eg, time, weather, and location), and interaction data (eg, information searched, liked, or rated) [1,5]. HRS can minimize the burden of delivering recommendations by only doing so when there is a real benefit (eg, when an individual is motivated and capable of following a recommendation) and can target multiple related behaviors at once (eg, diet, physical activity, and mental health). HRS have shown growth since 2012, particularly in the fields of lifestyle, such as personalized healthy nutrition recommendations, and personalized health promotion, such as recommending treatments of clinical diagnoses [2]. With increasing interest in using artificial intelligence to improve health promotion and prevention, and more than 50% (1 in 2 European Union citizens, aged 16-74 years) of individuals using the internet to find health information [6], the use of HRS, although still an emerging area, is expected to grow in digital health [7,8].

### The Need for a Health Equity Lens

However, digital health interventions, including HRS, are often not developed to target health inequities, “systematic differences in the health of groups and communities occupying unequal positions in society that are avoidable and unjust” [9]. For instance, HRS have scarcely been tested in populations that are marginalized, such as those belonging to low SES and racial and ethnically minoritized individuals [10]. This is a widespread issue, including in high-resource settings, with evidence from European countries and the United States showing that White individuals and those with higher education levels, income, and English proficiency tend to have higher access to and use digital health technologies more often, instead of the groups who would benefit from these solutions most [11]. There is a lack of attention to issues of equity and inclusion, and a tendency to develop from a technology-centered (instead of a human-centered) perspective in the HRS field [2].

### The Socioecological Model to Illustrate the Health Equity Potential of HRS

One way of working toward a perspective with an equity lens is the use of the socioecological model—a conceptual framework depicting spheres of influence over human behavior. This model is widely used in public health for scaling health promotion and implementation initiatives, to enable more effective public health interventions for complex social issues [12]. It has also been adapted for digital health equity [10,13]. It provides representations of how multiple, nested levels of influence (eg, community, institutional, and policy factors) interact in dynamic ways to shape individual health [14]. The socioecological model is based on a systems view of health—individual health is impacted by interactions with immediate peers and surroundings as well as broader institutional and societal norms [15]. It helps illustrate how HRS could address not just individual health needs but also the underlying conditions—such as access to health care, social support, and healthy lifestyles—that shape health outcomes. As far as we are aware, this framework has not been applied to HRS. Although some of these arguments can be applied to all digital health interventions, HRS have a unique role in the field because of their data-driven personalization and capacity to be strongly personalized due to profile- and context-crucial information.

Here we adapt the model to examine the unique potential of HRS to target and influence all its levels and thereby have great potential to promote health equity across society. We explain and illustrate how HRS can contribute to health equity by collecting data and targeting health at the systems level, integrating the context of individuals, families or peers, communities, and societies. We provide selected examples of previous work that could contribute to different layers of the framework and discuss challenges. Based on our analysis, we provide future research opportunities for HRS to target multiple levels of influence.

The novelty of this perspective is the intersection of recommender systems and public health, in particular health equity. HRS are still developing but starting to become integrated into medicine and public health [2]. There have been discussions about ethical considerations of recommender systems [16], but their potential on healthy equity, remains underdiscussed. With growing societal health inequities, and the World Health Organization’s recent call for equitable participation in a digital world [17], investing in equitable development of this new technology is essential now. As the field moves forward, we argue that an explicit health equity lens is needed from the start, not as an afterthought.

## Overview

**Figure 1:**
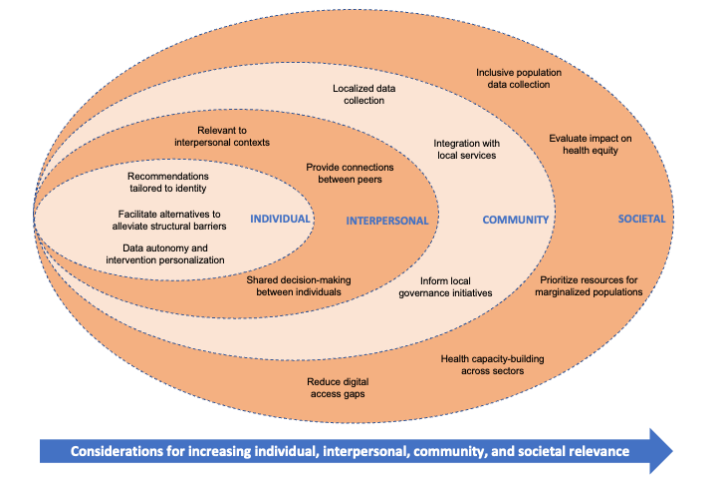
How health recommender systems can contribute to decreasing health inequities (illustrated within the socioecological model). Individual health is conceptualized as shaped by multiple levels, illustrated through concentric circles.

The paper is structured as follows. First, we illustrate the potential of HRS for decreasing health inequities based on the socioecological model (individual, interpersonal, community, and society; in “The potential of HRS using the socioecological model” section). Through an exploratory literature search, we provide selected examples of how HRS can contribute to health equity, by situating these examples into the different layers of the framework. We then investigate challenges and provide future research priorities for the HRS field based on the gaps we identify (“Recommendations for Research Priorities” section). Alongside Figure 1 [18], Table 1 presents implications of relevant factors that encompass each level and main actions for research priorities. We then provide a general conclusion.

**Table 1:** The potential of health recommender systems and main actions.

Socioecological level	The potential of HRS^a^	Main actions
Individual	Make tailored recommendations associated with identity (eg, healthy food associated with cultural traditions) Include alternative recommendations to overcome structural barriers (eg, based on budget and neighborhood safety) Allow individuals to determine what data they provide and which health domains (eg, physical, mental, and social) to focus on	Incorporate structural barriers to good health in personalization (social determinants of health) Centering intervention designs in the lived experience of the potential users (participatory design) Prioritize culturally appropriate interventions (eg, by considering decolonization of predominant approaches)
Interpersonal	Account for influence of family (eg, households) and social networks (eg, work environments) Leverage role and data on relationships (eg, connecting patients with similar challenges and demographics and peer endorsements) Provide opportunities for shared decision-making (eg, between patients and their caregivers)	Centering intervention designs in the lived experience of the potential users and other stakeholders (participatory design)
Community	Incorporate data on health factors relevant to a particular community (ie, localized data collection) Foster collaboration with local institutions (eg, grocery stores) to improve health equity (eg, access to fresh produce) Guide local governance initiatives for community health initiatives (eg, usage of parks)	Centering intervention designs in the lived experience of the potential users and other stakeholders (participatory design)
Societal	Address “health data poverty” through inclusive data collection (eg, marginalized populations Enable policy makers to build capacity between sectors to address social determinants of health through targeted interventions (eg, identifying key areas or populations in need of public health interventions)	Report and evaluate impact health inequities (eg, what disadvantages or privileges are perpetuated) Consistent and high-quality internet access Planning and delivery of HRS to marginalized populations Advocate for health equity policies based on HRS data

^a^HRS: health recommender systems.

## The Potential of HRS Using the Socioecological Model

The potential of HRS for decreasing health inequities based on the socioecological model (individual, interpersonal, community, and society) is described further in this study (Figure 1).

### Individual Level

The individual level includes individual personal characteristics, such as age, education, income, and health history [19]. Using individual-level data from phones and wearables, HRS can provide personalized suggestions that integrate and recognize structural barriers to good health that marginalized groups face more often. For example, for lower SES individuals, HRS for nutrition can combine budgetary restrictions with culture and familiarity motives (eg, food associated with cultural traditions, or a sense of safety and comfort), which are more important to individuals with fewer resources and diverse cultural identities [20]. For individuals living in areas where outdoor activities may be unsafe or impossible because of weather conditions, physical activity HRS could offer alternative options, such as indoor exercises, community-centered physical activities, or free web-based classes. Furthermore, HRS can also empower users through prioritizing user decisions over system performance, which is related to digital self-efficacy, and facilitates trust in HRS. For example, in a previous study, an HRS for health coaching allowed participants to pick their own health domain to work on, and only use the information they wanted to provide, such as not using sensors or answering all health-related questions [21]. Thus, the use of HRS can lead to more equitable health care recommendations and empowered health decisions surrounding different lifestyle factors (eg, physical, mental, and social).

### Interpersonal Level

The interpersonal level includes an individual’s closest social circle, such as friends, partners, family members, and colleagues, all of whom can influence their health behavior [19]. HRS has the potential to positively influence the health of families, households, work environments, and other small social networks by leveraging the data of interpersonal relationships [22]. For example, HRS can provide recommendations based on what worked for others with similar health [23]. HealthNet, a patient social network platform, used a recommender system to allow patients to find others with similar conditions, learn about what treatments were useful for them, and suggest health facilities or doctors consulted by patients with similar health status [24]. However, the demographic background of patients, and the incorporation of this information into the HRS, was not reported. Basing recommendations on others with similar socioeconomic and ethnic or racial minoritized backgrounds may make HRS even more effective. Furthermore, social media network data can be used, as shown by Oliva-Felipe and colleagues [25], who developed an HRS with educational content tailored to their specific profile of dementia caregivers. HRS could also leverage peer endorsements, allowing users to see recommendations or reviews from their social connections or members of a community with similar health goals [22]. Tapping into the power of social influence and community may specifically benefit lower SES individuals by making recommendations more relevant to them and trustworthy.

### Community Level

The community level includes the settings in which people work, live, and have social relationships, such as schools, workplaces, and neighborhoods, which affect health [19]. HRS can play a pivotal role in facilitating health promotion and preventive strategies tailored to the community-specific risk and protective factors by capturing data specific to a community (ie, localized data collection). For example, Wayman and Madhvanath [26] describe an HRS that makes dietary recommendations based on grocery receipt data. Leveraging this kind of data, HRS developers could collaborate with local businesses, such as grocery and convenience stores, to tailor the amount of fresh fruits and vegetables guided by food purchasing data [27]. HRS developers can also work with local governments to develop systems that allow low-income residents to receive recommendations on where to shop for healthy and affordable foods. The data collected from HRS can also be used to work with the city or county to identify the most suitable walking trails, parks, and indoor exercise sites and publicize these to the community. Thus, the ability of HRS to leverage localized data provides ample opportunities for community-level interventions.

### Societal Level

The societal level includes societal factors that influence health, such as the economic, educational, and social policies that help to create, maintain, or lessen health inequities [19].

HRS can contribute to the societal level by collecting data from individuals to build inclusive data sets with trends in health behaviors on a population level, which can be leveraged for policy making. Currently, individuals of lower SES and ethnic or racial marginalized individuals are systematically underrepresented in (open) health data sets (eg, the UK Biobank, with half a million UK participants) [28]. If HRS become widely used, by collecting data on the health of these marginalized populations in combination with contextual data, HRS can help to solve the problem of “health data poverty”, the inability of individuals, groups, or populations to benefit from discovery or innovation due to a scarcity of adequately representative data [29]. Policymakers can, for example, use these data to better pinpoint inadequate health care facilities, nutritional deficiencies, or insufficient infrastructure for healthy living and use this to devise social policies.

Regarding major public health issues like climate change, which disproportionately affects the health of marginalized populations [30], HRS could provide insight into human behavior that leads to high carbon emissions, such as food waste, or actions that can promote sustainable food consumption [31]. HRS could thus help policymakers allocate resources more efficiently by identifying areas or populations in need of specific public health interventions. This application of HRS aligns with the “Health in All Policies” (HiAP) approach promoted by the World Health Organization, in which policymakers in diverse sectors (eg, environment, agriculture, and urban planning) align efforts and health [32], consider the health impacts and benefits of plans to address employment, education, or housing. Furthermore, after implementing health policies, recommender systems could track their impact on health outcomes and provide policymakers with real-time feedback on the effectiveness of their policies [33]. Data insights collected through HRS could form the basis for more targeted policy interventions to reduce health inequities.

### Recommendations for Research Priorities

As we have described above in this section, HRS have immense potential to decrease health inequities. However, they are currently not designed from a health equity lens. Below we describe gaps in the current literature based on our analysis. For HRS to be more useful and accessible to marginalized populations, we need new design guidelines. Future research priorities are further described in this study that span several levels of the socioecological framework (also illustrated in Table 1).

### Incorporating Structural Barriers to Good Health in Personalization (Individual and Community)

Despite the potential of incorporating many data sources, as we explained above, most HRS do not integrate factors associated with the social determinants of health [34] and primarily use basic demographic information like age and gender, as well as individual health behaviors for tailoring recommendations [2]. As also mentioned by Richardson et al [10], in their digital health equity framework, this oversight could exacerbate health inequities, as marginalized individuals are less likely to benefit from interventions focused on behavioral factors, because of limited resources and competing priorities, which are greater in these populations. Future research should explore the integration of the social determinants of health into HRS, such as SES, healthy food scarcity, and digital and health literacy.

### Decolonial Perspectives in HRS Design (Individual, Interpersonal, and Community)

Because of their capability to incorporate diverse data sources, HRS are very suitable to be designed to contextualize, recognize, and incorporate diverse perspectives. Scientific knowledge in the digital health field is however currently predominantly produced through the lens of Western scholars, which marginalizes diverse and Indigenous health perspectives. In the case of HRS, users themselves have been scarcely involved in the design process. For example, in a recent review of 51 HRS, only 10 recruited users in the development of HRS testing, and the majority did not define the target group of the HRS intervention [2]. Furthermore, the majority of HRS studies are conducted in the United States and China, and low-income countries or low-resource settings are underrepresented [2].

Because of the underrepresentation of marginalized groups and lack of geographical diversity, HRS likely have cultural biases, which can make them less relevant to underrepresented groups. For example, mental health support integrated with religious or spiritual practices may be more accessible and relevant for some underrepresented groups, but it contrasts the body-mind separation of mental health dominant in a Western biomedical paradigm [35].

Decolonization involves critically re-evaluating interventions to remove cultural biases [36]. For example, mental health support integrated with religious or spiritual practices may be more accessible and relevant for some underrepresented groups, but it contrasts the body-mind separation of mental health dominant in a Western biomedical paradigm [35]. It involves centering intervention designs on the lived experience of the potential users. Thus users, especially those from marginalized backgrounds, should be included in the design and testing of HRS. This can be done through participatory research methods, where community members collaborate on all aspects of the project with researchers and other stakeholders, including feature design, testing, and dissemination [36]. This also involves examining power relationships that may underline the use of their technologies and structural and cultural factors that may broadly influence well-being [36]. Besides user involvement, technological collaborations with traditional health practitioners, such as herbalists or local healers [35] community, or faith-based organizations could help to understand and mitigate these power imbalances [37].

### Evaluating the Impact of HRS on Health Equity (Community and Societal)

HRS researchers and developers should critically evaluate and report how their solutions may impact health inequities (eg, what disadvantages or privileges the research may create) [38,39]. For example, successful implementation and deployment of HRS (and many other digital health interventions) at the community level depends on structural factors such as consistent and high-quality internet access, which may be particularly challenging for resource-constrained areas [10]. If data is not available in sufficient detail or quantities, this will impact the effectiveness of HRS, leading to their lower effectiveness for marginalized populations.

Furthermore, beyond the design phase, funding for the planning and delivery of HRS to marginalized populations is necessary, to avoid that successful HRS projects fail to scale up to maturity to impact health [40]. In addition, we also need the data to track the reach and effectiveness of HRS in decreasing health inequities (metrics beyond health outcomes) [41], such as access to and quality of care, and advocate for policy changes based on these data [42]. Addressing these structural challenges and advocating for inclusive research are essential steps toward ensuring the equitable deployment and effectiveness of HRS in marginalized communities.

## Conclusion

HRS have great potential in decreasing health inequities through accessible, personalized health promotion interventions, which can influence the individual, intrapersonal, community, and societal levels. HRS can leverage data from various sources to enable deep personalization of health promotion strategies based on the wider sphere of influence of health behavior and, through localized data collection, provide actionable insights for policymakers on population health. However, we argue that, with this emerging and developing field, health equity should be a priority, not an afterthought. Future research should focus on new design paradigms, including incorporating data on the social determinants of health, designing from a lens of decolonizing HRS, and evaluating the impact on health equity. Although this research does not provide definitive solutions, it lays a foundation for future studies, and we hope it benefits ongoing discourse in the field. With changes in practices and design with an intentional equity focus, HRS could play a crucial role in access to health promotion, improving the health of marginalized groups, and decreasing health inequities.

## Abbreviations

HiAP: Health in All Policies
HRS: health recommender systems
SES: socioeconomic status
